# Professional Recognition, Reality Shock and Nurses’ Well-Being After the Bologna Reform in Spain: A Qualitative Study of Key Informants’ Perspectives

**DOI:** 10.3390/healthcare14142146

**Published:** 2026-07-16

**Authors:** Alicia Méndez-Salguero, María García-Magán, Fernando Urcola-Pardo, Ana Belén Subirón-Valera

**Affiliations:** 1 General Surgery Unit, Central University Hospital of Asturias, Av. de Roma s/n, 33011 Oviedo, Spain; alimensal@gmail.com; 2 Department of Physiatry and Nursing, Faculty of Health Sciences, Universidad de Zaragoza, C/Domingo Miral s/n, 50009 Zaragoza, Spain; mgmagan@unizar.es (M.G.-M.); subiron@unizar.es (A.B.S.-V.); 3 SAPIENF Research Group (B53-23R), Universidad de Zaragoza, C/Pedro Cerbuna, 12, 50009 Zaragoza, Spain

**Keywords:** nurses’ well-being, reality shock, transition shock, nursing workforce, professional identity, competence recognition, healthcare system, nursing education, Bologna Process, Spain

## Abstract

**Highlights:**

**What are the main findings?**

- Nursing academic reform in Spain increased expectations regarding autonomy, competence recognition and professional development.
- Misalignment between university education and healthcare-system structures may contribute to Reality Shock and nurses’ well-being, highlighting the role of organizational and institutional factors beyond transition support programs.

**What are the implications of the main findings?**

- Aligning education, competence recognition, career development and nursing leadership may support retention and workforce sustainability.

**Abstract:**

**Background/Objectives**: The Bologna Process and the European Higher Education Area consolidated the transition of nursing education in Spain from diploma-level training to a university degree framework. Although previous research has examined newly graduated nurses’ transition to practice, less attention has been paid to how key informants involved in educational reform interpret its long-term professional implications, including their potential relevance for nurses’ well-being. This study explored key informants’ perspectives on the implications of the Bologna Process and the Nursing White Paper on nursing education and professional development in Spain. **Methods**: An exploratory qualitative study was conducted using in-depth semi-structured interviews with six key informants involved in nursing educational reform in Spain. Interviews were conducted between June and November 2025, audio-recorded, transcribed verbatim, and analyzed using reflexive thematic analysis. **Results**: Three main themes were identified: (1) academic professionalization and the generation of professional expectations; (2) structural misalignment between university-based professionalization and clinical practice; and (3) Reality Shock, professional identity and nurses’ well-being. **Conclusions**: From the perspective of key informants, Reality Shock may be understood not only as an individual transition difficulty, but also as a possible expression of structural misalignment between academic professionalization and healthcare organizations. These findings suggest that strengthening nurses’ well-being may require organizational strategies that translate educational reform into professional recognition, autonomy, leadership opportunities, and coherent career development.

## 1. Introduction

The implementation of the European Higher Education Area (EHEA) and the Bologna Process represented a major structural and pedagogical transformation in nursing education across Europe [1]. In Spain, this transition involved the shift from a diploma-based model to a university degree framework, with the aim of improving comparability between programs, promoting student mobility, and aligning training with competency-based, student-centered approaches [2]. The White Paper on Nursing [3] contributed to this process by defining the professional profile of nurses and establishing a set of competencies intended to guide curriculum development. This transformation was reinforced by European regulatory frameworks, particularly Directive 2005/36/EC [4], which established minimum training requirements for nurses responsible for general care and facilitated the recognition of professional qualifications across member states. These developments consolidated nursing as a graduate profession and enabled access to postgraduate education pathways, including master’s and doctoral programs [5].

Despite these advances at the academic and regulatory levels, the extent to which these reforms have translated into changes in professional practice, professional recognition and organizational conditions remains unclear and continues to be debated in the literature [6]. Previous research has consistently identified discrepancies between educational preparation and the demands of clinical environments, particularly during the transition from student to practicing nurse [7]. This transition period has been widely described as a critical stage in professional development, during which newly graduated nurses must integrate theoretical knowledge, clinical skills, and professional identity within complex organizational settings [8,9].

The concept of “reality shock”, originally introduced to describe the mismatch between expectations developed during training and the realities of professional practice, remains relevant in contemporary nursing contexts [10]. Later developments, particularly the concept of “transition shock”, have expanded this understanding by emphasizing the emotional, cognitive, sociocultural, and organizational challenges experienced by newly graduated nurses when entering clinical practice [11]. Recent reviews and primary studies confirm that this transition remains a critical period, often marked by stress, uncertainty, difficulties integrating theoretical knowledge into practice, role ambiguity, and the need for organizational support [8,12,13,14,15,16,17,18,19]. At the same time, the previous literature on European nursing education reforms has emphasized that the transition from education to professional practice should also be understood within broader educational and institutional contexts [20,21].

These transition experiences are relevant not only at the individual level, but also in relation to broader organizational outcomes, including job satisfaction, professional well-being, and workforce retention [12,16]. In recent years, increasing attention has therefore been paid to nurses’ well-being in the context of workforce shortages, high workload, burnout, intention to leave, and organizational pressures [9,13]. International reports further emphasize that nursing workforce sustainability depends not only on education capacity, but also on employment conditions, differentiated professional roles, leadership, continuing professional development, and mechanisms to support nurses’ mental well-being [22]. Recent evidence also suggests that leadership, career development, work well-being, and work–life balance may be relevant to nurse retention [23].

Recent European higher education reports show that the European Higher Education Area continues to prioritize convergence, quality assurance, recognition, mobility, and the comparability of qualifications [24]. However, in the Spanish nursing context, it remains unclear whether the academic professionalization promoted by the Bologna reform has been perceived as accompanied by equivalent changes in organizational recognition, autonomy, career development, and professional identity within healthcare institutions.

While previous research has explored the transition experience of newly graduated nurses, less attention has been paid to the perspectives of individuals who were directly involved in the design and implementation of educational reforms [8,18,19]. Key informants can provide insight into the intended objectives of the Bologna Process and the Nursing White Paper, the challenges encountered during implementation, and their perceived long-term implications for professional recognition, clinical practice, and nurses’ well-being. Therefore, this study aimed to explore key informants’ perspectives on how the Bologna Process and the Nursing White Paper have shaped nursing education and professional development in Spain. By examining these perspectives, the study seeks to contribute to a more nuanced understanding of how educational reforms may shape expectations regarding professional practice and how healthcare organizations may be relevant to nurses’ professional identity and well-being.

## 2. Materials and Methods

### 2.1. Study Design

This study adopted an exploratory qualitative design to examine perspectives on the implementation of the Bologna Process and the Nursing White Paper in Spain, particularly regarding their implications for professional practice and nurses’ well-being. A qualitative approach was considered appropriate to capture in-depth, experience-based insights from individuals directly involved in, or with extensive knowledge of, educational reform processes.

The reporting of this study was guided by the Consolidated Criteria for Reporting Qualitative Research (COREQ). The completed COREQ checklist is provided as Supplementary File S2.

Consistent with the use of reflexive thematic analysis, the study was informed by an interpretive qualitative orientation. From this perspective, participants’ accounts were understood not as direct representations of a single objective reality, but as situated interpretations shaped by their professional trajectories, institutional positions, and involvement in nursing educational reform. Accordingly, themes were understood as analytic constructions developed through the researchers’ active and reflexive engagement with the data.

### 2.2. Participants and Sampling

Participants were selected using purposive sampling according to their relevance to the study aim and their capacity to provide in-depth information about nursing educational reform in Spain. For the purposes of this study, key informants were defined as individuals with extensive and specialized knowledge of nursing education reform, particularly in relation to the transition from diploma-level nursing education to the Nursing Degree, the implementation of the Bologna Process, the Nursing White Paper promoted by ANECA, the Tuning Project, or the development of nursing degree curricula, academic management, or long-standing university teaching experience in Spanish universities.

Eligible participants were individuals with more than 20 years of professional academic or policy-related experience who had been directly involved in one or more processes related to nursing educational reform in Spain.

Sample adequacy was assessed in terms of information power rather than statistical representativeness [25]. The study aim was specific, participants had highly specialized knowledge of the phenomenon under study, and their professional trajectories were closely connected to the historical and institutional processes being analyzed. The interviews sought rich, contextually detailed accounts of the transition from diploma-level nursing education to the Nursing Degree and implications for professional development. The purpose of the study was to develop an in-depth interpretive understanding of key informants’ perspectives on the perceived implications of nursing educational reform in Spain.

### 2.3. Data Collection

Data were collected through in-depth semi-structured interviews conducted between June and November 2025. Four interviews were conducted face-to-face, three in Zaragoza and one in Oviedo, and two interviews were conducted online, depending on participant availability and logistical considerations. All interviews were conducted in Spanish and lasted between 40 and 90 min. Because the Bologna Process and the implementation of the Nursing Degree in Spain began years before data collection, the interviews were designed to elicit retrospective and historically informed reflections on the perceived long-term implications of these reforms, rather than contemporaneous accounts of their initial implementation.

The interviews were conducted by the doctoral researcher with two members of the research team. Brief handwritten notes were taken during the interviews to support contextual understanding and subsequent analysis.

A semi-structured interview guide was developed to explore key domains related to the implementation of the Bologna Process and the Nursing White Paper in nursing education. These domains included: (1) the impact of the Nursing White Paper on nursing education; (2) adaptation to the Bologna Process and European regulations; (3) challenges and evolution of the educational system; and (4) future perspectives for nursing education in Spain. The interview guide is provided as Supplementary File S1.

All interviews were audio-recorded with participants’ consent. Audio recordings were transcribed using Pinpoint and subsequently reviewed and corrected manually by the doctoral researcher to ensure accuracy. Quotations selected for publication were translated from Spanish into English by the doctoral researcher and reviewed by the research team, including an additional team member who contributed to translation and linguistic revision, to ensure that the translated excerpts preserved the meaning, tone, and contextual sense of the original accounts.

### 2.4. Data Analysis

Data were analyzed using reflexive thematic analysis, following the approach developed by Braun and Clarke [26,27]. This method was selected due to its flexibility and suitability for identifying patterns of meaning within qualitative data while allowing for an interpretative and theoretically informed analysis.

The analysis was supported by ATLAS.ti for data management and coding. An inductive approach was adopted, whereby initial codes were generated from the data rather than imposed a priori. The analysis primarily focused on semantic patterns of meaning, while also considering underlying patterns where relevant to the study aim.

The analytical process followed the six phases outlined by Braun and Clarke: (1) familiarization with the data through repeated reading of transcripts; (2) generation of initial codes across the dataset; (3) searching for potential themes by grouping related codes; (4) reviewing and refining themes in relation to the dataset; (5) defining and naming themes; and (6) producing the final report.

Coding and theme development were conducted collaboratively by the research team. The doctoral researcher led the initial analytical process, while other members of the research team participated in coding and theme refinement. Preliminary codes, candidate themes, and interpretative decisions were discussed in team meetings, with particular attention to alternative interpretations and to the coherence between themes, coded extracts, and the overall dataset. These discussions were aimed at enhancing depth of interpretation rather than achieving inter-coder reliability, in accordance with the principles of reflexive thematic analysis.

In line with a reflexive approach, themes were understood as interpretative constructions developed through active engagement with the data, rather than as entities that emerge passively from it. Analytical notes and working documents were used throughout the process to support the development, comparison, and refinement of codes and themes.

### 2.5. Rigor and Reflexivity

Methodological rigor was addressed through transparency in the research process, systematic engagement with the data, collaborative analysis, and reflexive consideration of the researchers’ positionality. In accordance with reflexive thematic analysis, rigor was not understood as the achievement of inter-coder reliability, but as the coherence between the research question, methodological approach, analytical process, and the credibility and richness of the interpretations developed.

The research team included clinical and academic nurses. Researchers contributed an academic perspective informed by experience in nursing education, curriculum development, research, and university structures. The team had also conducted previous work on nursing curricula in Spanish universities, which had raised questions about variability in nursing education programs and the historical transition from diploma-level to degree-level nursing education. This background contributed to the development of the research questions, but it also required the team to remain attentive to the possibility of interpreting participants’ accounts through pre-existing assumptions about academic professionalization, professional recognition, and the alignment between education and practice.

Some members of the research team had prior professional or academic relationships with several participants, whereas other participants were not previously known to the doctoral researcher. These relationships were considered during data collection and analysis. To support reflexivity, preliminary codes, candidate themes, and interpretative decisions were discussed within the research team. Particular attention was paid to whether interpretations were sufficiently grounded in the data, whether alternative explanations could be considered, and whether the analysis distinguished participants’ accounts from the researchers’ own assumptions about nursing education and professional development.

### 2.6. Ethical Considerations

The project was authorized by the Data Protection Unit of the University of Zaragoza and was reviewed and approved by the Research Ethics Committee of Aragón (CEICA) (C.P.-C.I. PI25/520). The study was conducted in accordance with the ethical principles of the Declaration of Helsinki [28] and with applicable Spanish and European regulations on personal data protection.

All participants received written and verbal information about the aims and procedures of the study, the voluntary nature of participation, the right to withdraw or discontinue participation at any time, the audio recording of interviews, the confidential handling of data, and the use of anonymized information for scientific publication. Written informed consent (Appendix A) was obtained from all participants before data collection. In reporting the findings, participants were identified using codes, and potentially identifying information was removed or presented in broad categories to preserve confidentiality.

## 3. Results

### 3.1. Profile of Key Informants

A total of six participants were included in the study: five nurses and one physician with extensive expertise in nursing education, academic reform, and professional policy. All participants had more than 20 years of professional or academic experience. The sample included both active and retired professionals and comprised three women and three men. Participants’ profiles included involvement in the Nursing White Paper, the Tuning Project, nursing degree curriculum design, academic management, and university teaching in both public and private university contexts. This composition allowed the study to include perspectives from individuals involved in different stages and dimensions of nursing educational reform.

The characteristics of the six key informants are presented in Table 1.

### 3.2. Main Themes

Three main themes were developed through reflexive thematic analysis: (1) academic professionalization and the generation of professional expectations; (2) structural misalignment between academic professionalization and healthcare system realities; and (3) Reality Shock, professional identity, and nurses’ well-being.

These themes should not be understood as isolated descriptive categories, but as connected dimensions of a broader interpretive account.

#### 3.2.1. Academic Professionalization and the Generation of Professional Expectations

Key informants interpreted the implementation of the European Higher Education Area (EHEA) and the transition from the Diploma to the Nursing Degree as a turning point in the academic professionalization of nursing in Spain. The reform was generally described in positive terms, not only as a structural modification of university education, but also as an opportunity to strengthen academic recognition, professional development, and disciplinary autonomy.

Interviewees emphasized that the Nursing Degree positioned nursing within a university framework as other academic disciplines. This fact was perceived as helping to overcome some of the historical limitations associated with the former Diploma. Access to a complete academic pathway—including undergraduate, master’s and doctoral education—was described as one of the most significant changes introduced by the reform, as it enabled opportunities for academic, research and professional development that had previously been limited. As one informant explained:

*“It opened the doors for us, as if to say: you can go as far as you want”.*

(E6)

The Bologna reform was also described as “a tremendous advance” (E5) and “a great opportunity for nursing” (E6), particularly in relation to academic harmonization and European convergence. Informants highlighted that the new educational model promoted greater standardization of training, facilitated the mobility of students and faculty members, and reinforced the international dimension of nursing education.

Another positively valued aspect was the extension of nursing education to four academic years. Informants did not interpret this change merely as a longer training period, but rather as an opportunity to consolidate knowledge, incorporate new competencies, and support greater professional maturity among future nurses. As one informant stated:

*“It is a very necessary year, because nurses really did not have enough time to consolidate their knowledge (…) new generations are emerging that are much more mature, much more grounded, and more confident in their approaches and in the role they have to perform”.*

(E1)

Informants also perceived that the implementation of the Degree increased the presence of nurse educators within universities and strengthened nursing perspectives within nursing education itself. This was viewed as progress compared with more medically dominated educational models, as informants perceived that it enabled nursing-specific knowledge, values, and interpretative frameworks to become more visible within university teaching. As one informant noted:

*“It is nurses themselves who are now participating much more in teaching and in the undergraduate education of future nursing professionals”.*

(E3)

#### 3.2.2. Structural Misalignment Between Academic Professionalization and Healthcare System Realities

Despite the positive assessment of the Nursing Degree, informants described a persistent tension between the academic progress achieved by nursing and the conditions of professional practice within the healthcare system. Their accounts suggested that university reform had contributed to training nurses with greater competence, critical thinking, and opportunities for academic development. However, this progress was not always perceived as having translated into equivalent occupational, institutional, or organizational recognition.

Informants framed the problem not as a lack of preparation among newly graduated nurses, but as a limited capacity of the healthcare system to recognize and integrate the competencies promoted during university education. Several accounts described healthcare structures that continue to operate according to organizational logics predating the implementation of the Nursing Degree, where nurses are still frequently positioned in instrumental, technical or subordinate roles. One informant expressed this tension as follows:

*“The healthcare system does not want nurses; it is not organized for nurses and considers nurses simply as workforce. What difference does their qualification make?”.*

(E1)

The interviews also pointed to what informants perceived as an underuse of professional competencies. Although nurses were considered more highly educated, informants indicated that many professional settings did not provide sufficient opportunities to apply advanced knowledge, exercise autonomy or assume responsibilities consistent with their educational level. One informant summarized this idea through a former student’s experience:

*“Once, a former student told me: ‘you train us far beyond what the system allows us to be’”.*

(E4)

The interviews further suggested that academic reform was not accompanied by equivalent transformations in job structures, leadership models or professional development pathways. According to the interviewees, healthcare organizations were perceived as continuing to demand profiles adapted to earlier professional models. This perceived disconnect was captured in the following statement:

*“The university creates the nurse of the future. And in the system, she finds the nurse of the past”.*

(E1)

This misalignment was described not simply as a gap between theory and practice, but as a broader tension between university-based professionalization and the organizational reality of healthcare systems. Informants suggested that healthcare organizations do not always provide conditions that enable nurses to practice with the autonomy, responsibility, and professional identity development promoted during university education:

*“The problem is not that universities have to train nurses for the current reality of care. Rather, the reality of care itself has to change, because nurses have acquired greater competencies. So, this is still a problem we have.”*

(E4)

The accounts also suggested that this misalignment may have implications for professional well-being. Professional distress was not described only in relation to workload or the demands of clinical work. According to the key informants, nurses’ well-being appeared closely linked to recognition, autonomy, competence development, and the possibility of providing care consistent with professional values. This tension was described as particularly visible during the transition from university education to clinical practice, when expectations generated during training confronted organizational realities perceived as more restrictive. One informant described this experience as follows:

*“There are nurses who are trained and who believe that nursing is one thing, and when they go to work… they encounter reality, the wall they crash directly into”.*

(E4)

Taken together, the accounts suggest that the main limitation identified was not the educational reform itself, but the lack of correspondence within professional practice.

#### 3.2.3. Reality Shock, Professional Identity, and Nurses’ Well-Being

Within this theme, key informants interpreted Reality Shock as the encounter between the professional expectations generated by academic professionalization and healthcare settings perceived as insufficiently aligned with those expectations. Informants’ accounts suggested that this misalignment may be relevant not only to organizational conditions, but also to nurses’ professional identity. They described how university education promoted professional autonomy grounded in evidence-based care and independent clinical judgment. However, these expectations could be constrained by work environments that continued to position nursing as a technical and subordinate role:

*“A system that treats people in the same way regardless of the education they have completed and the capacities and competencies they have acquired is a system that does not treat nurses well.”*

(E4)

Within this context, the interviews pointed to a tension between the professional identity promoted through university education and the position nurses were perceived to occupy within healthcare structures. This discrepancy was associated, in the informants’ accounts, with feelings such as frustration, disenchantment, and loss of professional meaning. A recurrent issue in the interviews concerned the perceived invisibility of the nursing role. Informants suggested that nursing has historically struggled to make itself visible through its own language and professional self-representations. According to the interviews, the transition from the Diploma to the Degree was not perceived as having fully resolved this issue, as some forms of recognition and professional representation continued to reflect earlier conceptions of nursing. As one informant explained:

*“Well, the change from one thing to another had its advantages and its disadvantages. And for me, the greatest disadvantage is that we were not able to use clear language that could make us visible”.*

(E5)

Informants also linked this perceived invisibility to the persistence of strongly medicalized organizational structures where its preserved the old academic hierarchies, and a secondary position within many decision-making spaces, both in healthcare organizations and universities. One interviewee expressed this perception as follows:

*“I doubt that we will ever rid ourselves of the stigma of being a second-class or lesser profession, not because of what we do on a daily basis, but because of university studies themselves”.*

(E3)

The perceived absence of specific spaces for nursing leadership and decision-making also emerged as a relevant issue. Informants described difficulties in accessing positions related to management, planning and organizational influence, despite the academic development achieved by the profession. As one informant stated:

*“We were in a power structure; we were very low down in the chain of power in universities (…) and what was nursing? Well, it was nothing”.*

(E1)

Informants further emphasized that perceived invisibility may particularly affect the relational and emotional dimensions of nursing care, which require time, recognition, and appropriate organizational conditions to be adequately developed:

*“We need time and sometimes private spaces, which we do not have, to work on that part of invisible care, don’t we? The emotional part”.*

(E2)

From this perspective, nurses’ well-being was interpreted as closely connected to recognition, professional identity, and meaning at work. The accounts suggested that when nursing is reduced to a predominantly technical or task-oriented role, opportunities to experience work as a source of professional growth, disciplinary contribution, and fulfillment may become limited. Overall, the interviews pointed to a structural tension between an educational model that promotes autonomy, competence development, and disciplinary identity, and healthcare environments that do not always provide sufficient support for nursing competencies in practice.

## 4. Discussion

The findings of this study suggest that the implementation of the Nursing Degree in Spain, within the framework of the European Higher Education Area (EHEA) and the Nursing White Paper, was perceived by key informants as a relevant step forward in the academic professionalization of nursing. Informants associated this reform with greater opportunities for competence development, academic progression, and professional recognition. However, their accounts also suggested that these educational and regulatory advances were not always accompanied by equivalent changes in the occupational, organizational, and institutional conditions of professional practice. This interpretation should not be read as evidence that the Bologna Process itself produced these tensions. Rather, Bologna provides the historical and institutional context through which key informants reflected on issues that may also have preceded the reform or may be linked to broader organizational, professional, and labor-market conditions [6,20].

From this perspective, the misalignment identified should not be understood solely as a gap between theory and practice, nor as an exclusively educational or curricular issue. Previous research suggests that when professional development encounters work environments in which the nursing role continues to be perceived as technical, subordinate, or insufficiently differentiated, professional well-being may be affected [22,23,29,30]. Thus, from the informants’ perspective, nurses’ well-being appears to be linked not only to workload or occupational stress, but also to recognition, autonomy, professional identity, meaning at work, retention, and the sustainability of the nursing workforce.

### 4.1. Academic Professionalization, Expectations, and the Limits of Educational Reform

The positive assessment of the transition from the Diploma to the Degree is consistent with the European literature on the impact of the Bologna Process on nursing education. The incorporation of nursing into the framework of the European Higher Education Area (EHEA) promoted the comparability of educational programs, the reorganization of initial training and access to postgraduate academic pathways, thereby contributing to the consolidation of nursing within the university sphere [7,31]. In this regard, the findings of this study align with previous research highlighting the role of the Bologna Process in the modernization and harmonization of nursing education across Europe. Davies described this transition as a “silent revolution” in European higher education, emphasizing its contribution to educational convergence, academic and professional mobility, and the consolidation of nursing within the university context [32]. However, the author also warned that these reforms could generate important tensions, particularly in relation to the economic, political and organizational barriers involved in consolidating a fully graduate and professionally recognized nursing workforce.

Nevertheless, academic harmonization does not necessarily imply an equivalent transformation in professional recognition. Although the Degree expanded nurses’ educational, research, and teaching opportunities, the informants’ accounts suggest that these advances have not always translated into an effective redefinition of the nursing role within the healthcare system. This distinction is particularly relevant because it helps to differentiate between academic professionalization and effective professionalization. The former refers to the advancement of nursing within the university; the latter requires that this advancement produce tangible changes in work organization, professional career pathways, clinical autonomy, and decision-making spaces. The perspectives of the key informants suggest that bridging the gap between academic and effective professionalization may require greater alignment between educational reforms and the organizational development of healthcare systems, so that the competencies fostered through university education can be effectively translated into professional practice.

From this perspective, the transition to the Degree involved not only an academic reorganization, but also the generation of expectations regarding the development of a more autonomous, reflective and research-oriented professional identity grounded in evidence-based practice [33]. Precisely for this reason, the absence of sufficient correspondence within the workplace may intensify perceptions of misalignment. The educational reform made it possible to imagine a more autonomous, research-oriented and academically consolidated nursing profession. However, according to the informants, the healthcare system did not always generate sufficient conditions for this professional profile to fully develop. Previous studies have similarly suggested that clinical environments are not always prepared to integrate newly qualified nurses and the competencies promoted during university education [20,21,34]. Overall, the perspectives of the key informants suggest that the Bologna reform may have made these tensions more visible within a graduate-level professionalization framework, rather than creating them by itself [21]. The challenges identified therefore concern not only educational reform, but also how healthcare systems, employers, and workforce policies translate educational advances into professional recognition, autonomy, and organizational conditions that enable nurses to fully develop their professional role [22,23,29].

### 4.2. Reality Shock as an Expression of Structural Misalignment

The concept of Reality Shock, originally described by Kramer, refers to the impact experienced when the values, expectations and learning acquired during education collide with the realities of professional practice [10]. Duchscher later expanded this perspective through the concept of Transition Shock, incorporating emotional, physical, intellectual, sociocultural and role-related dimensions associated with newly graduated nurses’ entry into the workplace [11]. Within this framework, the transition into professional practice can be understood not only as an individual adaptive process, but also as an experience shaped by the organizational conditions of healthcare systems.

The findings of this study engage with this literature while offering a complementary interpretation from the perspective of key informants. This interpretation helps avoid an exclusively individualizing understanding of Reality Shock. The difficulty may not lie solely in newly graduated nurses’ difficulties adapting to the workplace, but also in the limited capacity of healthcare organizations to incorporate the professional profile that universities have contributed to developing. When healthcare systems continue to operate according to organizational logics that predate the implementation of the Degree, the tension may occur not only between personal expectations and clinical reality, but also between two different models of nursing: one promoted through university education and another still embedded within certain institutional and organizational dynamics.

From this perspective, Reality Shock may be interpreted as an indicator of structural and institutional incoherence between the competencies promoted by educational reform and the actual opportunities to exercise them in clinical settings. The previous literature has already suggested that educational reforms alone are insufficient when healthcare environments are not adequately prepared to integrate newly qualified nurses and the competencies associated with graduate-level education [20,21,34]. Universities may train professionals with expanded competencies, critical thinking skills, research orientation and expectations of professional autonomy, while healthcare systems do not always provide sufficient opportunities for autonomous practice, differentiated responsibilities, participation in decision-making or coherent professional development pathways. Consequently, this disconnection may reduce the impact of educational reform and contribute to the perception that academic professionalization represents a promise only partially fulfilled. This interpretation is also consistent with broader European debates on future challenges for nursing education and the need to align educational development with changing professional and healthcare demands [35].

### 4.3. Nurses’ Well-Being: Autonomy, Recognition, and Professional Meaning

The findings of this study suggest that professional distress may not derive solely from workload or burnout. This interpretation is particularly relevant within broader conceptualizations of workplace well-being. Fisher argues that well-being at work extends beyond job satisfaction or the presence of positive emotions, encompassing dimensions related to growth, meaning, motivation, relationships, and personal and professional development [36]. Applied to the present findings, the transition to the Degree not only transformed the curricular structure of nursing education, but also generated expectations linked to eudaimonic dimensions of well-being, including competence development, autonomy, recognition, and professional projection.

The recent literature similarly suggests that nurses’ well-being is shaped by multiple individual, relational and organizational factors, including workload, working conditions, leadership, interpersonal relationships, professional recognition and opportunities for development [29]. From this perspective, nurses’ well-being cannot be understood solely as the absence of stress or burnout, but rather as the result of work environments that enable nurses to exercise their competencies, feel professionally supported, and maintain a coherent and recognized professional identity.

Consequently, when the academic professionalization promoted by the Degree is not accompanied by equivalent transformations in clinical settings, the misalignment described by the informants may be relevant to nurses’ professional well-being. This interpretation is also consistent with the literature on burnout and work engagement, which has shown how demanding work environments with limited support may negatively affect professional commitment and increase emotional exhaustion [37]. In Spain, recent reports by the General Council of Nursing and the Ministry of Health have highlighted concerns regarding care pressure, emotional distress, workforce retention and the limited occupational translation of nurses’ educational development, particularly in relation to specialist recruitment, professional career pathways and role differentiation [38].

Therefore, the risk of leaving the profession should not be interpreted solely as an individual or generational difficulty in adapting to clinical work. Rather, it may reflect the tension between an education that promotes autonomy, competence development, and professional identity, and healthcare systems that do not always provide sufficient conditions for these expectations to be fulfilled. This interpretation is consistent with studies linking retention and intention to leave to factors such as leadership, career development, professional dissatisfaction, and limited professional recognition [23,30]. Thus, nurses’ well-being should also be understood as a matter of professional sustainability. When nurses are unable to develop their competencies or build recognized professional trajectories, the issue may transcend individual distress and become an organizational challenge affecting retention, workforce planning, and the sustainability of healthcare systems.

### 4.4. Professional Identity, Invisibility, and Institutional Recognition

The findings suggest that the implementation of the Degree may have contributed to strengthening a more autonomous, academically grounded, and professionally self-aware nursing identity. However, this identity does not always appear to find an equivalent translation into decision-making spaces within the healthcare system. This tension may be interpreted from a historical perspective. The incorporation of nursing into the university system in Spain has previously been described as a process of academic normalization and professional identity construction [39]. Nevertheless, the informants’ accounts suggest that academic consolidation has not fully eliminated historical dynamics of subordination, medicalization or dependence on other disciplines. Consequently, nursing may have advanced in terms of education and academic legitimacy without this necessarily leading to an equivalent transformation of its position within healthcare structures.

The limited visibility and institutional voice of nursing appear to extend beyond symbolic representation, and may also be reflected in the distribution of power, leadership and decision-making capacity. Limited access to management positions, reduced participation in certain planning structures and difficulties in establishing differentiated professional pathways may restrict the profession’s ability to influence organizational transformation and the development of nursing practice. From this perspective, professional recognition cannot be understood solely in terms of social appreciation, but also in relation to the real capacity to participate in decision-making processes and shape the profession’s own field of practice.

In this regard, the paradox between social recognition and institutional under-recognition is particularly relevant. The 2024 Spanish Health Barometer identifies nurses as among the healthcare professionals most highly valued by the general population [40]. However, the informants described a limited occupational, organizational and political translation of this recognition. This contradiction may contribute to professional dissatisfaction, as recognition, autonomy, and opportunities for professional development are closely related to broader conceptions of well-being at work that emphasize meaning, personal growth, and fulfillment in professional practice [36].

Taken together, these findings suggest that professional identity and nurses’ well-being are closely related to organizational recognition and institutional positioning. Academic professionalization may strengthen expectations of autonomy, visibility and professional development, but these expectations may remain only partially fulfilled when healthcare structures do not provide equivalent opportunities for leadership, recognition and professional participation.

### 4.5. Implications for Education, Healthcare Organization, and Professional Retention

The findings of this study have educational, organizational and workforce-related implications. First, they suggest that continuing to improve nursing education is necessary, but not sufficient. The academic professionalization achieved through the implementation of the Degree may be more effectively consolidated if it is accompanied by equivalent developments in working conditions, professional career pathways, competence recognition, and opportunities for nursing leadership. From this perspective, the implications of educational reform extend beyond academic settings and are also relevant to workforce planning, professional retention and healthcare system sustainability.

Second, the findings suggest that alignment between universities and healthcare systems may be understood as a strategy for promoting professional well-being. This involves not only adapting curricula to labor market demands, but also ensuring that the competencies promoted during education can be meaningfully enacted in clinical practice. Achieving this alignment may require greater coherence between educational models, professional roles, advanced practice opportunities, specialist recruitment, research development, clinical leadership and participation in decision-making processes.

Third, this study supports a less individualizing interpretation of Reality Shock. When the misalignment between expectations and professional reality is understood exclusively as an adaptation problem among newly graduated nurses, responses tend to focus primarily on induction programs, mentoring or emotional support. Although these strategies are important, they may be insufficient if the structural conditions that generate the misalignment remain unchanged. Addressing Reality Shock may therefore require not only support during professional transition, but also healthcare organizations capable of recognizing and effectively integrating nurses’ competencies.

Finally, these findings have implications for professional retention and workforce sustainability. Limited recognition, restricted autonomy, and reduced opportunities for professional development may contribute not only to dissatisfaction and burnout, but also to disengagement and intentions to leave the profession [23,30,37]. Consequently, promoting nurses’ well-being may require organizational conditions that enable nurses to practice in ways that are consistent with their education, professional identity, and disciplinary values.

Taken together, the findings suggest that improvements in nursing education alone may not guarantee equivalent improvements in professional experience or workplace well-being. Although the implementation of the Degree strengthened the academic legitimacy of nursing, its perceived implications for nurses’ well-being appear to be closely related to the healthcare system’s capacity to translate academic professionalization into effective recognition, autonomy, leadership, and professional development. In this regard, aligning university education, professional competencies and actual conditions of practice may represent a key strategy for strengthening nurses’ well-being, workforce retention and the long-term sustainability of healthcare systems. Achieving such alignment is likely to require not only educational reform, but also healthcare policies and organizational frameworks that recognize and support the competencies associated with graduate nursing education. Accordingly, the practical implications of this study should be located primarily at the level of healthcare-system governance and workforce planning, rather than at the level of curriculum reform alone [22,23,38]. Addressing the tensions described by key informants may require policies related to role differentiation, career pathways, working conditions, leadership opportunities, and participation in decision-making [22,29,38].

### 4.6. Study Limitations

This study has several limitations that should be considered when interpreting the findings. First, the study included a small sample of six key informants. Although this sample was considered appropriate for an exploratory qualitative study based on information power, it limits the breadth of perspectives represented. The findings are therefore not intended to be statistically generalized to the nursing profession as a whole or to all individuals and institutions involved in the implementation of nursing educational reform in Spain. However, the aim of the study was not to quantify the prevalence of the phenomenon or to achieve representativeness, but to develop an in-depth interpretive understanding of how individuals with extensive academic, professional, and policy-related experience perceived the relationship between educational reform, professional recognition, clinical practice, Reality Shock, and nurses’ well-being.

Second, the analysis has a retrospective dimension. The interviews were conducted in 2025, whereas the Bologna Process, the Nursing White Paper, and the transition from diploma-level nursing education to the Nursing Degree had begun years earlier. Participants’ accounts may therefore have been influenced by the subsequent evolution of university and healthcare systems, as well as by interpretations developed through accumulated professional experience.

Third, transcripts were not returned to participants for comment or correction, and participant-checking of the final themes was not conducted. However, analytical rigor was supported through systematic engagement with the data, collaborative coding and theme development, team discussions, and reflexive consideration of the researchers’ positionality.

Fourth, this study explored the Bologna Process from the perspectives of key informants who had been directly involved in nursing educational reform in Spain. Consequently, it does not provide a comparison with the views of nurses educated before and after the implementation of the European Higher Education Area (EHEA), recently graduated nurses, healthcare managers, employers, or other stakeholders involved in nursing education and professional practice.

Finally, this study was not designed to establish causal relationships between educational reform, Reality Shock, and nurses’ well-being. Rather, it offers a qualitative interpretation of key informants’ perspectives on how the perceived alignment—or misalignment—between university education and healthcare structures may be relevant to professional recognition, autonomy, professional identity and well-being.

## 5. Conclusions

The implementation of the Bologna Process and the Nursing White Paper in Spain was perceived by key informants as an important step in the academic professionalization of nursing, expanding educational opportunities, facilitating access to postgraduate education and strengthening the discipline’s university-based identity. However, the findings of this study suggest that these reforms were not always accompanied by equivalent changes in professional practice, particularly regarding competence recognition, professional autonomy, career development, and nurses’ participation in decision-making processes.

Within this context, Reality Shock may be understood not only as an individual experience associated with the transition into professional practice, but also as a possible expression of broader structural tensions between university education and the organizational conditions of healthcare systems. From the perspective of nurses’ well-being, this misalignment appears especially relevant, as it may affect dimensions related to professional meaning, recognition, identity, autonomy, and the possibility of practicing in accordance with the competencies acquired during education.

Consequently, promoting nurses’ well-being may require moving beyond curricular reform alone. Greater alignment between university education, professional roles, institutional recognition, advanced practice opportunities, career development, and nursing leadership may help to translate academic professionalization into meaningful conditions of professional practice. In this regard, strengthening coherence between educational reforms and healthcare system structures may be relevant not only to nurses’ professional well-being, but also to workforce retention and the long-term sustainability of healthcare systems.

Future research should build on these findings by comparing the perspectives of key informants with those of nurses educated before and after the Bologna reform, recently graduated nurses, healthcare managers, employers, and policy-makers. Such comparative research could contribute to a deeper understanding of how educational reform, organizational conditions, and labor-market factors may interact in shaping professional recognition, professional identity, autonomy, and nurses’ well-being, particularly in relation to meaning at work, professional fulfillment, and identity in clinical practice.

## Figures and Tables

**Table 1:** Anonymized characteristics of key informants.

Participant	Gender	Professional Profile	Status	Academic Experience	Institutional Background	Reform-Related Expertise
E1	Male	Physician academic	Retired	>30 years	Public university	Nursing White Paper coordination
E2	Female	Nurse academic	Retired	>30 years	Public university	Tuning Project
E3	Male	Nurse academic and healthcare professional	Active	>20 years	Public and private university	Nursing degree curriculum design
E4	Male	Nurse academic	Retired	>30 years	Public university	Nursing White Paper
E5	Female	Nurse academic with academic management and leadership experience	Active	>30 years	Public university	Nursing White Paper and curriculum development
E6	Female	Nurse academic	Retired	>30 years	Public university	Nursing White Paper

All participants had university teaching experience. Broad categories are used to preserve participant confidentiality.

## Data Availability

The data presented in this study are not publicly available due to privacy and confidentiality restrictions related to the qualitative interviews. Additional information may be available from the corresponding author upon reasonable request, subject to ethical and confidentiality considerations.

## Supplementary Materials

The following supporting information can be downloaded at: https://www.mdpi.com/article/10.3390/healthcare14142146/s1. Supplementary File S1: Semi-structured interview guide; Supplementary File S2: COREQ checklist.

## Abbreviations

The following abbreviations are used in this manuscript:

EHEA	European Higher Education Area
RS	Reality shock
SNS	Spanish National Health System
ANECA	National Agency for Quality Assessment and Accreditation

## Appendix A. Informed Consent Form for Interview

Principal Investigator: Alicia Méndez Salguero
  Institution/Affiliation: University of Zaragoza
  Contact: alimensal@gmail.com

Purpose of the study:

The purpose of this study is to explore perspectives on the impact of the Nursing White Paper, the Bologna Process and the evolution of nursing education in Spain.

Interview procedure:

Participation consists of an interview lasting approximately 45 min. The interview will explore the participant’s perspective on nursing education, professional recognition and future challenges.

Audio recording and use of information:

The interview will be audio-recorded to ensure accuracy and facilitate transcription. The recording will be used only for transcription and research purposes. Only the research team will have access to the recordings and transcripts.

Voluntary participation:

Participation is voluntary. Participants may refuse to answer any question or withdraw from the study at any time without any negative consequences.

Consent:

I confirm that I have read and understood the information provided. I agree to participate in this study. I authorize the audio recording of the interview and the use of the information provided for scientific research and subsequent publication.

Participant signature: [              ]
  Researcher signature: [              ]
  Date: [              ].
