# Supplementary material for: Professional Recognition, Reality Shock and Nurses’ Well-Being After the Bologna Reform in Spain: A Qualitative Study of Key Informants’ Perspectives
Source: Healthcare (Basel). 2026 Jul 16;14(14):2146. doi: 10.3390/healthcare14142146 (PMC13410286; doi:10.3390/healthcare14142146)
Supplement: Supplementary file 1 [file healthcare-14-02146-s001.zip › healthcare-4395666_Supplementary File S1_InterviewGuide.pdf]

Supplementary File S1. Semi-structured interview guide

Title: Interview with key informants on the implementation of the Bologna Process in nursing education

Objectives

- To evaluate the impact of the Nursing White Paper on nursing education in Spain.
- To analyze how the Nursing White Paper was adapted to changes derived from the Bologna Process and European regulations.
- To obtain perspectives on the future of nursing education in Spain and its alignment with international standards.

1. Impact of the Nursing White Paper on nursing education
2. What fundamental changes did the Nursing White Paper promote in 2004 in nursing curricula in Spain?
3. How did the transition from diploma-level nursing education to the Nursing Degree affect the quality of education and the professional integration of nurses?
4. Adaptation to the Bologna Process and European regulations
5. What role has the Bologna Process played in the evolution of nursing education in Spain and its alignment with European standards?
6. In your view, did the first Nursing Degree programmes generally meet the requirements of the European Higher Education Area and Directive 2005/36/EC?
7. Challenges and evolution of the educational system

At present, nursing education is again facing a context of change, due to new regulations, including the 2024 Royal Decree on the organization of university education, changing population needs to which nursing must adapt, and the substantial variability in nursing curricula identified in analyses conducted before the COVID-19 pandemic.

1. What have been the main challenges in implementing this reform in universities, and what lessons have been learned?
2. What specific and transversal competences were key in the new Nursing Degree?
3. Future perspectives
4. Which aspects of the Nursing White Paper should be updated to better respond to the current challenges of the nursing profession?
5. What role should universities, professional nursing bodies, and health administrations play in the continuous improvement of nursing education?
