# Supplementary material for: Professional Recognition, Reality Shock and Nurses’ Well-Being After the Bologna Reform in Spain: A Qualitative Study of Key Informants’ Perspectives
Source: Healthcare (Basel). 2026 Jul 16;14(14):2146. doi: 10.3390/healthcare14142146 (PMC13410286; doi:10.3390/healthcare14142146)
Supplement: Supplementary file 1 [file healthcare-14-02146-s001.zip › healthcare-4395666_Supplementary File S2_COREQ.pdf]

Supplementary File S2. COREQ checklist

Nº	Item	Guide questions/Description	Reported on
Domain 1: Research team and reflexivity			
	Personal characteristics		
1	Interviewer/facilitator	Which author/s conducted the interview or focus group?	Methods, Section 2.3 Data collection
2	Credentials	What were the researcher's credentials? E.g. PhD, MD	Methods, Section 2.5 Rigor and reflexivity
3	Occupation	What was their occupation at the time of the study?	Methods, Section 2.5 Rigor and reflexivity
4	Gender	Was the researcher male or female?	Methods, Section 2.5 Rigor and reflexivity
5	Experience and training	What experience or training did the researcher have?	Methods, Sections 2.4 Data analysis and 2.5 Rigor and reflexivity
	Relationship with participants		
6	Relationship established	Was a relationship established prior to study commencement?	Methods, Section 2.5 Rigor and reflexivity
7	Participant knowledge of the interviewer	What did the participants know about the researcher? e.g. personal goals, reasons for doing the research	Methods, Section 2.3 Data collection
8	Interviewer characteristics	What characteristics were reported about the interviewer/facilitator? e.g. Bias, assumptions, reasons and interests in the research topic	Methods, Section 2.5 Rigor and reflexivity
Domain 2: Study design			
	Theoretical framework		
9	Methodological orientation and Theory	What methodological orientation was stated to underpin the study? e.g. grounded theory, discourse analysis, ethnography, phenomenology, content analysis	Methods, Sections 2.1 Study design and 2.4 Data analysis
	Participant selection		

10	Sampling	How were participants selected? e.g. purposive, convenience, consecutive, snowball	Methods, Section 2.2 Participants and sampling
11	Method of approach	How were participants approached? e.g. face-to-face, telephone, mail, email	Methods, Section 2.2 Participants and sampling
12	Sample size	How many participants were in the study?	Methods, Section 2.2 Participants and sampling; Table 1
13	Non-participation	How many people refused to participate or dropped out? Reasons?	Methods, Section 2.2 Participants and sampling
	Setting		
14	Setting of data collection	Where was the data collected? e.g. home, clinic, workplace	Methods, Section 2.3 Data collection
15	Presence of non-participants	Was anyone else present besides the participants and researchers?	Methods, Section 2.3 Data collection
16	Description of sample	What are the important characteristics of the sample? e.g. demographic data, date	Methods, Section 2.2 Participants and sampling; Table 1
	Data collection		
17	Interview guide	Were questions, prompts, guides provided by the authors? Was it pilot tested?	Methods, Section 2.3 Data collection; Supplementary File S1
18	Repeat interviews	Were repeat interviews carried out? If yes, how many?	Methods, Section 2.3 Data collection
19	Audio/visual recording	Did the research use audio or visual recording to collect the data?	Methods, Section 2.3 Data collection
20	Field notes	Were field notes made during and/or after the interview or focus group?	Methods, Section 2.3 Data collection
21	Duration	What was the duration of the interviews or focus group?	Methods, Section 2.3 Data collection
22	Data saturation	Was data saturation discussed?	Methods, Section 2.2 Participants and sampling
23	Transcripts returned	Were transcripts returned to participants for comment and/or correction?	Methods, Section 2.3 Data collection
Domain 3: analysis and findings			
	Data analysis		

24	Number of data coders	How many data coders coded the data?	Methods, Section 2.4 Data analysis
25	Description of the coding tree	Did authors provide a description of the coding tree?	Methods, Section 2.4 Data analysis; Results section
26	Derivation of themes	Were themes identified in advance or derived from the data?	Methods, Section 2.4 Data analysis
27	Software	What software, if applicable, was used to manage the data?	Methods, Section 2.4 Data analysis
28	Participant checking	Did participants provide feedback on the findings?	Methods, Section 2.3 Data collection
	Reporting		
29	Quotations presented	Were participant quotations presented to illustrate the themes/findings? Was each quotation identified? e.g. participant number	Results section
30	Data and findings consistent	Was there consistency between the data presented and the findings?	Results section
31	Clarity of major themes	Were major themes clearly presented in the findings?	Results section
32	Clarity of minor themes	Is there a description of diverse cases or discussion of minor themes?	Results section
